# Supplementary material for: Dietary supplementation with copper nanoparticles influences the markers of oxidative stress and modulates vasodilation of thoracic arteries in young Wistar rats
Source: PLoS One. 2020 Feb 21;15(2):e0229282. doi: 10.1371/journal.pone.0229282 (PMC7034852; doi:10.1371/journal.pone.0229282)
Supplement: S1 File — (DOCX) [file pone.0229282.s001.docx]

- Cu supplementation enhances lipid peroxidation compared to Cu deficient diet.
- Nano Cu increases thiols and decreases carbonyl groups compared to ionic Cu.
- Nano Cu potentiates the vascular response induced by the gasotransmitters NO and CO.
- iNOS contributes to the vasodilation in nano Cu supplemented rats.
- cGMP/PKG signaling cascade is involved in the vascular regulation of nano Cu.
